# ^210^Po Log-normal distribution in human urines: Survey from Central Italy people

**DOI:** 10.1080/15376510902740978

**Published:** 2009-06-30

**Authors:** D. Sisti, M. B. L. Rocchi, M. A. Meli, D. Desideri

**Affiliations:** 1 Dipartimento S.U.A.N. Polo Scientifico complesso ex-Sogesta, Loc.Crocicchia, University of Urbino ‘Carlo Bo’, Urbino, Italy; 2 Institute of General Chemistry, University of Urbino ‘Carlo Bo’, Urbino, Italy

**Keywords:** Polonium-210, urine, upper reference limits, Log-normal distribution

## Abstract

The death in London of the former secret service agent Alexander Livtinenko on 23 November 2006 generally attracted the attention of the public to the rather unknown radionuclide ^210^Po. This paper presents the results of a monitoring programme of ^210^Po background levels in the urines of noncontaminated people living in Central Italy (near the Republic of S. Marino). The relationship between age, sex, years of smoking, number of cigarettes per day, and ^210^Po concentration was also studied. The results indicated that the urinary ^210^Po concentration follows a surprisingly perfect Log-normal distribution. Log ^210^Po concentrations were positively correlated to age (*p* < 0.0001), number of daily smoked cigarettes (*p* = 0.006), and years of smoking (*p* = 0.021), and associated to sex (*p* = 0.019). Consequently, this study provides upper reference limits for each sub-group identified by significantly predictive variables.

## Introduction

The poisoning incident in London during November 2006 involving a lethal intake by Mr Alexander Livtinenko of ^210^Po, presumably via ingestion, sparked renewed interest in the field of ^210^Po toxicity to humans (Editorial 2007; Harrison et al. 2007; Stather 2007]).

^210^Po occurs widely in nature and is an important component of man's natural radiation background (Parfenov 1974). The main route of ^210^Po intake by the human body is the ingestion with food stuffs, other studies reported that smoking also represents a significant route (Parfenov 1974; Skwarzec 2001; Khater 2004; Arifi et al. 2006). Ingestion with drinking water, especially of underground origin, represents another route of ^210^Po intake. Inhalation of ^222^Rn released from the soil also contributes in ^210^Po body burden. The radiation exposure, delivered to sensitive tissues, as that of air pathways in the respiratory system, may induce cancer both alone and synergistically with non-radioactive carcinogens. The absorption coefficient of ^210^Po into blood from the digestive tract was estimated to be 35%, and the excretion of this radionuclide from the body in the urine was 14–15-times less than feces (Scott and West 1975; Fellman et al. 1994). However, the body burden of ^210^Po in a normal human body may differ from one person to another, depending upon such as diet habits, origin of drinking water, residence place which effects radon exposure rate, and also smoking habits like smoking rate, smoking period, and the type of smoking material. Therefore, many factors may affect ^210^Po intake and lead to a variation in body burden in different individuals (Parfenov, 1974; UNSCEAR 1988; Editorial 2007; Harrison et al. 2007; Stather 2007).

Due to the variability in the estimates of ^210^Po content among different populations, the prospective of this article is limited to the evaluation of the background level of ^210^Po concentration in the urines of non-contaminated people living in an area of Central Italy (near the Republic of S. Marino) where the Urbino University is located. In fact, in this area, specific sources of ^210^Po (industrial and military nuclear centrals, fall-out of nuclear experiments) are absent.

The analytical method used is capable of measuring natural levels of ^210^Po in many types of sample, including urine, and it requires 2–3 days from receipt of a 24-h sample. This method was used by the Radiation Protection Division of the Health Protection Agency (HPA) after the poisoning incident in London to assess the level of ^210^Po in urine of members of the public and various employees who may have had contact with a contaminated person or location (Health Protection Agency 2007). It is also a rapid method for dose assessment purposes. In fact, from the amount of ^210^Po excreted in the urine, by well-established methods, it is possible to calculate the amount of ^210^Po that originally entered the body, and, hence, using the appropriate dose conversion factors, the resulting radiation dose.

Scientific literature doesn't provide estimations of the statistical distribution of ^210^Po concentration in human urines, although this information is necessary to establish unbiased Upper Reference Limits (URL). The goal of this human study is to address this needed information.

## Materials and methods

### Sampling

One hundred and thirty-two samples of urine were analyzed of people living in the area of Central Italy (near to the Republic of S. Marino, Adriatic coast) where the Urbino University is located.

All subjects agreed to participate in the study voluntarily after receiving–each of them individually–a formal explanation of the aims and of the structure of the study; they participated voluntarily as unpaid subjects in the experiment, and provided written informed consent prior to participation in the study. The study was in accordance with the Declaration of Helsinki, and was approved by the ethical board of the University of Urbino.

People were chosen taking into account the following variables: age, sex, and smoking habits (years of smoking, number of cigarettes per day).

One hundred and thirty-two subjects, (5–84 years old, 59 males and 73 females, 39 cigarette smokers and 67 non-smokers) participated in this study. The continuous smoking individuals were identified as smokers, whereas irregularly smoking individuals were excluded from this study. The subjects classified as non-smokers have never smoked during their life. Subject characteristics are reported in Table 1.

**Table 1:** Descriptive statistics of sample.

	Min	Max	Mean (SD)	*n*
*Smokers*				
Age	19	75	43.3(16.2)	
Years of smoking	1	60	23.9(14.3)	
N.cigarettes/day	5	40	14.3(7.6)	
Sex				
Males				19
Females				20
*Non-smokers*				
Age	5	84	37.3(23.8)	
Sex				
Males				40
Females				53

### Radioanalytical method

The urine of 24 h were collected in the plastic 2–l bag. After addition to 0.5 L of urine of a known quantity of ^209^Po as internal standard yield tracer and of 50 ml of conc. HNO_3_, the sample was stirred and heated on a hot plate at 150°C, evaporated until 25 ml, and transferred to a suitable beaker. Sequential treatments with conc. HNO_3_ and H_2_O_2_ were performed to destroy the organic material and release free polonium ions into the solution. Then the solution was evaporated to dryness and the residue was treated three times with conc. HCl at a controlled temperature of 85–90°C to ensure complete nitrate removal. Finally the residue was dissolved with warming in 120 ml of 1 M HCl and filtered.

Polonium was deposited at a controlled temperature of 85–90°C and at pH 1.5–2.0 continuously for 4 h in a silver disk, placed in a syringe and immersed into 200 mL of 1MHCl solution containing 10 ml of 20% hydroxylamine hydrochloride and 10 ml of 25% sodium citrate. The silver disk was measured by α-spectrometry. No preliminary separation was required and essentially quantitative recoveries were obtained by using a standard ^209^Po tracer (Jia et al. 2000; Desideri et al. 2007; Murray et al. 2007).

As ^210^Po emits alpha particles at 5.407 MeV, polonium source was counted using a high resolution alpha ray spectrometry system with silicon detectors (Canberra, USA) for 86,000 s. The mean counting efficiency was 31.7 ± 3.1% and the background was ∼ 2·10^−6^ s^−1^ in the energy region of interest. A motor-driven vacuum pump provided adequate evacuation of the vacuum chambers of the system. The mean chemical yield was 60.1 ± 14.2% and the detection limit resulted to be 0.2 mBq/d.

The uncertainties given with the final results are 1 SD, resulting from the propagation of all random counting uncertainties occurring anywhere in the entire measurement process.

A validation check of the method was carried out by the participation in an international inter-comparison organized by the International Atomic Energy Agency (IAEA, Vienna).

### Statistical analysis

Analysis of covariance (ANCOVA) was performed to evaluate the relationship between three predictive quantitative variables (age, years of smoking, number of cigarettes per-day), one predictive factor (sex), and ^210^Po extraction rate as dependent variable (Shaeskin 2000).

Normality and homoscedasticity, which are required for the application of parametric methods, were checked and resulted to be substantially improved by Log transformation of ^210^Po extraction rate, as graphically confirmed by the p-p plot drawn before and after Log transformation. Moreover, to test if ^210^Po extraction rate came from a normally distributed population, the Shapiro-Wilks test was performed, both on raw and on Log-tranformed data. In addition, the 95th 97.5th, and 99th percentile upper reference limits (URL) of ^210^Po extraction rate were determined basing on Log-normal distribution. Statistical analysis was performed using the Statistical Package for Social Sciences (SPSS) and Excel (Microsoft® software).

## Results

The gender proportions between the smoking habit groups (non-smokers vs smokers) were similar, but there was a slight difference in age distribution, with a higher dispersion of nonsmokers vs smokers age (Table 1). The results showed a higher Po-210 extraction rate in the smokers group (Table 2).

**Table 2:** ^210^Po extraction rate (mBq/d) in smokers and non-smokers groups (minimum and maximum value, mean and SD).

Po-210 (mBq/d)	Min	Max	Mean (SD)
Smokers	1.3	73.5	11.7(13.5)
Non-smokers	0.45	58.3	9.8(10.6)

^210^Po extraction rate in smokers, regardless of age and number of cigarettes, ranged from 1.3–73.5 mBq/d in urine, with a mean value of 11.7 ± 13.5 mBq/d.

**Figure 1:**
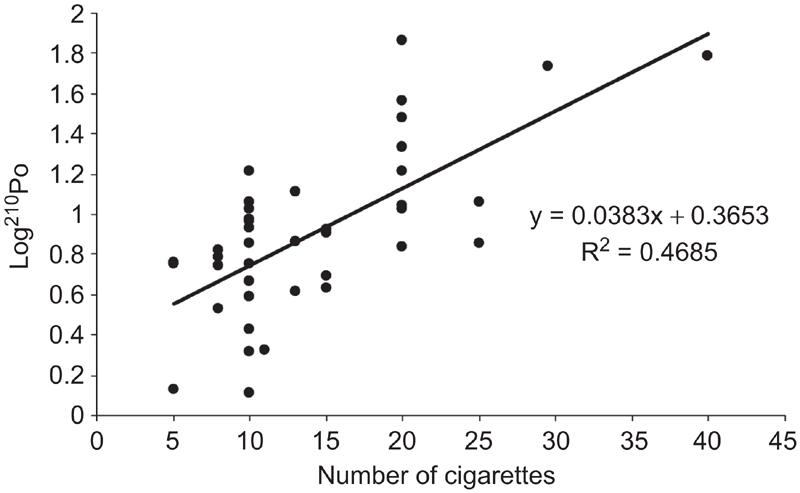
Correlation between daily average number of cigarettes smoked and Log ^210^Po extraction rate (mBql/d) (*p* < 0.01).

Non-smokers, regardless of age, showed activity levels ranging from 0.45–58.3 mBq/d, with a mean value of 9.8 ± 10.6 mBq/d. As expected, ^210^Po extraction rate in smokers significantly depended on number of cigarettes per day (Pearson's *r* = 0.68; *p* < 0.01), with the following exponential relation: ^210^Po = 0.365*10^0.038* n cigarettes^ (Figure 1).

Some studies (Parfenov 1974; Shabana et al. 2000) indicated that ^210^Po and ^210^Pb have long been associated with tobacco plants. Based upon the mean values these results reflect a slight, but significant, correlation of ^210^Po in the smoker's urinary excretion. This fact is due, probably, to the many factors, in addition to smoke, that may affect the ^210^Po intake and lead to the variation in the ^210^Po content of the urines for different individuals; moreover ^210^Po intake from smoking depends on smoking habits, in fact, smoking rate, smoking period, and type of smoking material can play an important role in ^210^Po intake. In this research the majority of smokers smoked a very low number of cigarettes per day: for 89.8% of smokers the number of cigarettes per day ranged between 5–20.

**Figure 2:**
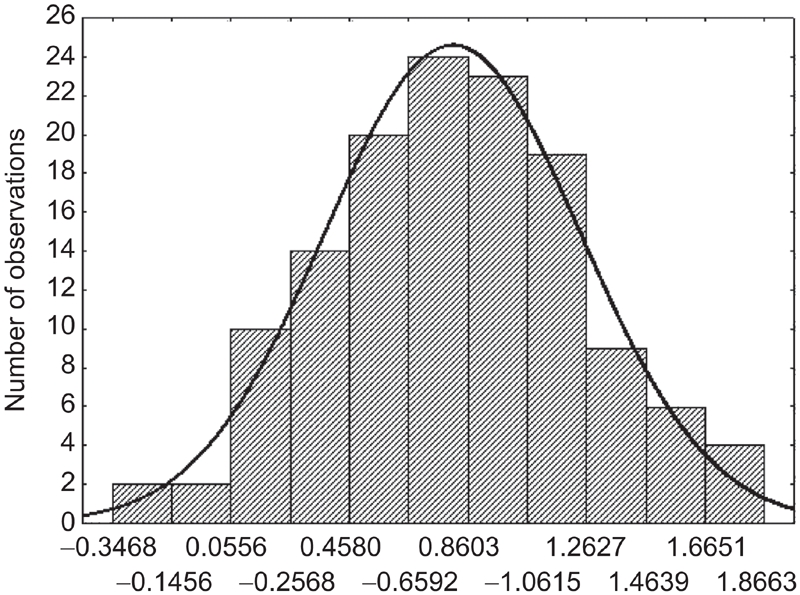
Distribution of Log ^210^Po extraction rate (mBq/d); in bold line the estimated normal distribution.

**Figure 3:**
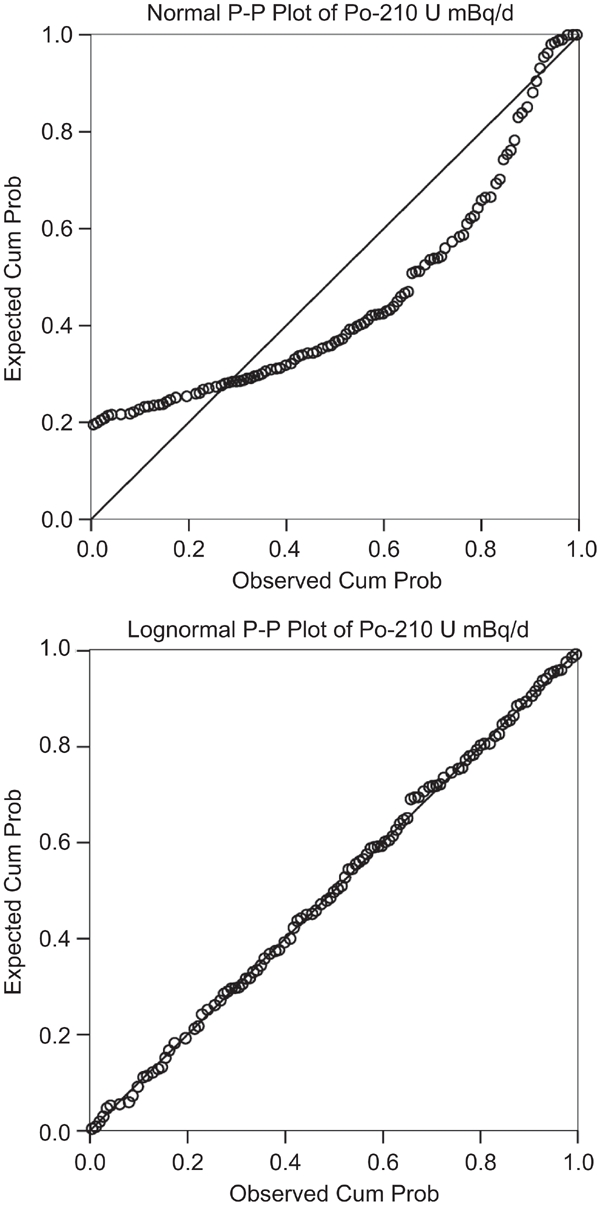
P-P plot of ^210^Po extraction rates (raw data and Logtransformed data).

Normality of distribution of ^210^Po extraction rate was strongly biased (*p* < 0.0001, Shapiro-Wilks test); on the contrary, Log-transformed data showed a clear normal distribution (*p* = 0.983, Shapiro-Wilks test) (Figure 2). Normal distribution of Log-tranformed data is confirmed by P-P plots (Figures 3A and B).

The ANCOVA model shows significant effects both for sex (*p* = 0.019) and for all the considered covariates; Log ^210^Po extraction rates were strongly positively correlated to age (*p* < 0.0001), mean number of daily smoked cigarettes (*p* = 0.006) and years of smoking (*p* = 0.021) (Table 3).

**Table 3:** ANCOVA results considering Log ^210^Po extraction rate as depending variable.

Factor and covariates	df	F	Sig.
Years of smoking	1	5.44	0.021
Number of cigarettes	1	7.72	0.006
Age	1	23.29	0.000
Sex	1	5.69	0.019
Error	128		
Total	133		

*R*^2^ adjusted (*R*^2^_adj_) value reports the global ANCOVA results, expressed as percentage of the total sum of squares explained by the factor and covariates; *R*^2^_adj_ = 0.211 showed a low proportion of observed variability; other not considered variables, such as environmental radiation, life and diet habits, origin of drinking water, residence place which effects radon exposure rate, and other subjective behaviour, could be further contamination sources.

Parfenov (1974) reports that children excrete only half as much ^210^Po as adults. Besides, between the ages of 16–60, the ^210^Po concentration, scarcely but significantly, changes. This could be due to the fact that ^210^Po intake with ingestion is similar for adults, whereas it could be lower for young people.

**Figure 4:**
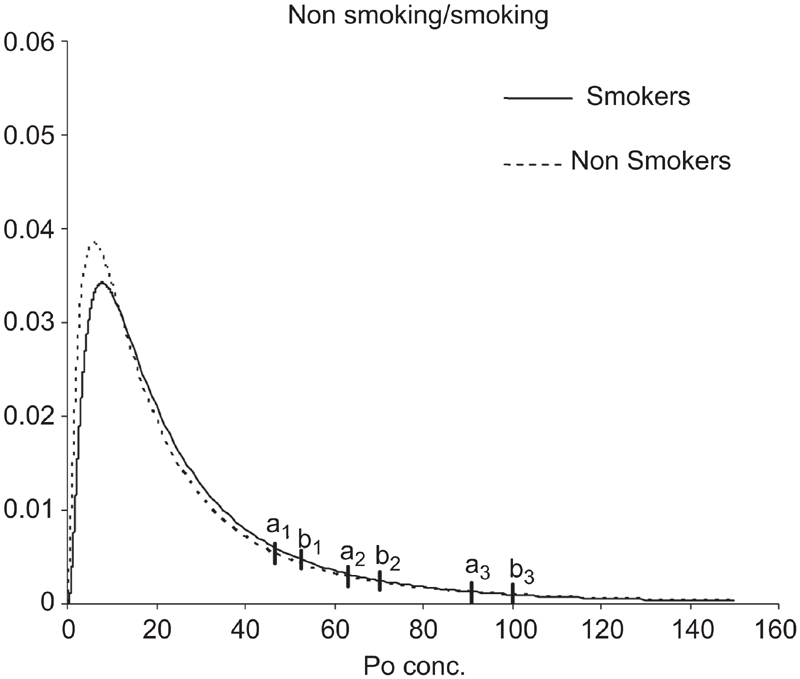
Lognormal estimated distributions. Vertical bars indicate the 95th, 97.5th, and 99th percentiles of URL for non-smokers (a1, a2, a3) and smokers (b1, b2, b3) sub-samples.

**Figure 5:**
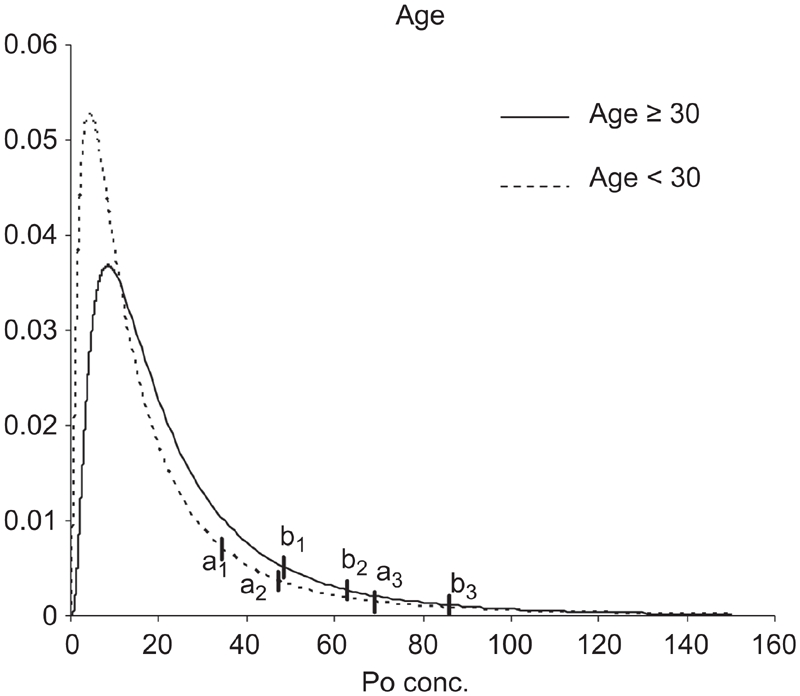
Lognormal estimated distributions. Vertical bars indicate the 95th, 97.5th, and 99th percentiles of URL for age < 30 (a1, a2, a3) and age ≥ 30 (b1, b2, b3) sub-samples.

**Figure 6:**
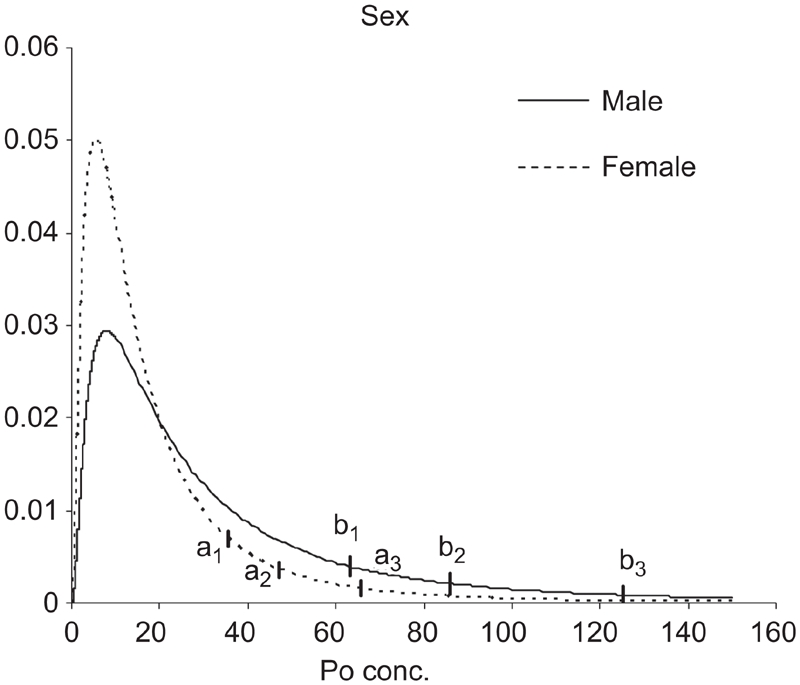
Lognormal estimated distributions. Vertical bars indicate the 95th, 97.5th, and 99th percentiles of URL for female (a1, a2, a3) and male (b1, b2, b3) sub-samples.

This study has also defined the URL for ^210^Po concentration in urine of people living in Central Italy, according to results of ANCOVA results; in Figures 4–6, Log-normal distributions were plotted for two factors (sex and smoking habit) and a covariate (age), recoding in a binary factor (age <30; age ≥30).

The URL was calculated as the 95th, 97.5th, and 99th percentile of the ^210^Po concentration; for the smokers subsample 95th, 97.5th, and 99th percentiles were 52.5, 70.3, and 100.3 mBq/d, respectively; for the non-smokers sub-sample they were 46.6, 63.1, and 90.9 mBq/d (Figure 4).

For the age ≥30 sub-sample, 95th, 97.5th, and 99th percentiles were 48.6, 62.9, and 86.0 mBq/d, respectively; for the age <30 sub-sample they were 34.4, 47.2, and 69.0 mBq/d (Figure 5).

Finally, for the male sub-sample, 95th, 97.5th, and 99th percentiles were 63.1, 86.1, and 125.5 mBq/d, respectively; for the female sub-sample they were 35.7, 47.1, and 65.9 mBq/d (Figure 6).

## Discussion

This paper presents the results of the monitoring program on the urines of non-contaminated people living in an area of Central Italy (near the Republic of S. Marino) to evaluate the background level of the ^210^Po concentration in this region Approximately 93.9% of the whole sample resulted in < 30 mBq/d. In the monitoring program of HPA (UK) of March 2007 on 741 urine samples tested, 81.0% were < 30 mBq/d. HPA has judged this level of 30 mBq/d to be one at which there will certainly be some activity above the natural background ^210^Po level, which typically falls in the range of ∼ 5–15 mBq/d (HPA 2007). Moreover, these results are very similar to those obtained by other authors in Italy: 14.3 ± 16.0 and 9.1 ± 3.8 for people living in regions having a high and a low radioactivity background, respectively (Mancini et al. 1984).

Several studies indicated that ^210^Po has long been associated with tobacco plants; moreover ^210^Po intake from smoking depends on smoking habits; in fact, smoking rate, smoking period, and the type of smoking material can play an important role in ^210^Po intake. In this research the majority of smokers smoked a very low number of cigarettes per day: for 89.8% of smokers the number of cigarettes per day ranged between 5–20. However, in this study, ^210^Po in smokers is exponentially correlated with number of cigarettes per day; for number of cigarettes < 10, ^210^Po level is similar to non-smokers, while ^210^Po level significantly increases for number of cigarettes > 20 (Figure 1). Data show that ^210^Po concentration is higher for males than for females, whereas it is significantly different between smokers and non-smokers. This is the first study, to our knowledge, which has reported a ^210^Po level difference in urine in relation to sex; this feature could depend on life-style differences and could not be in relation to an actual difference in metabolism depending on sex. Further investigations ought to be carried out in other countries in order to verify this issue.

In this study, for the first time the shape distribution of urinary ^210^Po excretion has been described; unexpectedly, the resultant Log-normal distribution showed a perfect goodness of fit with observational data (*p* = 0.983). This finding allowed us to accurately define the URLs of urinary ^210^Po excretion, both for the general population and for stratified sub-groups (sex, age, smoking habit). Confidence interval embraces values which are not significantly different from the expected value; this kind of information should be particularly useful to identify subjects presenting a urinary ^210^Po level significantly higher than expected values, referred to each specific stratum of population. If the urinary ^210^Po level of one subject is higher than URL, it does not necessarily mean it is clinically important, but it does indicate at least an abnormal exposition of ^210^Po. Therefore, URLs of urinary ^210^Po excretion may be used for screening exposed subjects, in order to assess possible ^210^Po contamination.

## References

[b1] Al-Arifi M. N., Alkarfi K. M., Al-Suwayeh S. A., Aleissa K. A., Shabana E. I., Al-Dhuwaili A. A., Hassan M. I. (2006). Levels of ^210^Po in blood, urine and hair of some Saudi smokers. J. Radioanal. Nucl.Chem..

[b2] Desideri D., Meli M. A., Feduzi L., Roselli C., Rongoni A., Saetta D. (2007). ^238^U, ^234^U, ^226^Ra, ^210^Po, concentrations of bottled mineral waters in Italy and their dose contribution. J. Environ. Radioactivity.

[b3] (2007). Editorial. Radiation protection and international intrigue. Radiat. Protect. Dosimetry.

[b4] Fellman A., Ralston L., Hickman D., Ayres L., Cohen N. (1994). Polonium metabolism in adult female baboons. Radiat. Res..

[b5] Harrison J., Legget R., Lloyd D., Phipps A., Scott B. (2007). Polonium-210 as a poison. J. Radiol. Protect..

[b6] Health Protection Agency (2007). Update statement on the public health issues related to Polonium-210. http://www.hpa.org.uk/hpa/news/articles/pressreleases/2006/251106pol210htm.

[b7] Jia G., Belli M., Blasi M., Marchetti A., Rosamilia S., Sansone U. (2000). ^210^Pb and ^210^Po determination in environmental samples. Appl. Radiat. Isotopes.

[b8] Khater A. E. M. (2004). Polonium-210 budget in cigarettes. J. Environ. Radioactivity.

[b9] Mancini L., Renzetti A., Santori G. (1984). Livelli di polonio-210 nelle urine di alcuni gruppi della popolazione italiana. ENEA Report.

[b10] Murray M., Chang-Kyu K., Martin P. (2007). Determination of ^210^Po in environmental materials: a review of analytical methodology. Appl. Radiat. Isotopes.

[b11] Parfenov Yu D. (1974). Polonium-210 in the environment and in the human organism. Atom. Energy Rev..

[b12] Scott L. M., West C. M. (1975). Excretion of ^210^Po oxide following accidental inhalation. Health Phys..

[b13] Shabana E. I., Adb Elaziz M. A, Al-Arifi M. N., Al-Dhawailie A. A., Al-Bokari M. M.-A. (2000). Evaluation of the contribution of smoking to total blood polonium-210 in Saudi population. Appl. Radiat. Isotopes.

[b14] Shaeskin D. J. (2000). Handbook of parametric and non parametric statistical procedures.

[b15] Skwarzec B., Strumininska D. I., Ulatowski J., Golebiowski M. (2001). Determination and distribution of ^210^Po in to-bacco plants from Poland. J. Radioanal. Nucl. Chem..

[b16] Stather J. W. (2007). The Polonium-210 poisoning in London. J. Radiat.Protect..

[b17] United Nations Scientific Committee on the Effect of Atomic Ra-275 diation (1988). Sources, effects, and risk of ionising radiation. UNSCEAR 1988, Report to the Generally Assembly.

